# UFMylation: A Potential Modification for Neurological Diseases

**DOI:** 10.2174/011570159X340639240905092813

**Published:** 2025-01-02

**Authors:** Guanglu Che, Xiao Xiao, Tingyu Li, Jingdong Li, Linbo Gao

**Affiliations:** 1 Department of Laboratory Medicine, Key Laboratory of Birth Defects and Related Diseases of Women and Children, Ministry of Education, West China Second University Hospital, Sichuan University, Chengdu, Sichuan, 610041, P.R. China;; 2 Laboratory of Molecular Translational Medicine, Center for Translational Medicine, Key Laboratory of Birth Defects and Related Diseases of Women and Children (Sichuan University), Ministry of Education, West China Second University Hospital, Sichuan University, Chengdu, Sichuan 610041, P.R. China;; 3 Department of Medical Genetics, West China Second University Hospital, Sichuan University, Chengdu, Sichuan 610041, P.R. China;; 4 Institute of Hepato-Biliary-Pancreatic-Intestinal Disease, Affiliated Hospital of North Sichuan Medical College, Nanchong, Sichuan 637100, P.R. China

**Keywords:** UFMylation, UFM1, UBA5, UFC1, UFL1, neurological diseases

## Abstract

Neurological disorders are the leading health threats worldwide, characterized by impairments in consciousness, cognition, movement, and sensation, and can even lead to death. UFMylation is a novel post-translational modification (PTM) that serves as an important regulatory factor, promoting the complexity of protein structures and enhancing the diversity and specificity of functions. In UFMylation, ubiquitin-fold modifier 1 (UFM1) is covalently transferred to the primary amine of a lysine residue on the target protein through the synergistic action of three enzymes: the activating enzyme E1 of UFM1, the coupling enzyme E2 of UFM1, and the ligase E3. UFMylation has been proven to be involved in various cellular processes, such as the maintenance of genome homeostasis, autophagy, signal transduction during antiviral responses, cell death, and differentiation. Additionally, a growing number of evidence suggests that polymorphisms in genes related to the UFMylation system are associated with the risk of epileptic encephalopathy, microcephaly, neurodegenerative diseases, and schizophrenia. Therefore, the concept, enzymatic cascade, and biological functions of UFMylation are carefully summarized, along with its potential role in neurological diseases.

## 1 INTRODUCTION

The precursor protein generated directly by ribosomes and endoplasmic reticulum lacks of biological activity, which can become a functional mature protein after a series of post-translational processing known as post-translational modification (PTM). During PTMs, chemical groups or other proteins are attached to the specific amino acid residues of proteins, altering the spatial conformation or chemical properties of the proteins and promoting the diversification and specificity of their functions. There are many types of PTMs of proteins, such as phosphorylation, glycosylation, methylation, acetylation, ubiquitination, and various ubiquitin-like modifications [1]. One such ubiquitin-like modification is UFMylation, which involves the covalent binding of ubiquitin-fold modifier 1 (UFM1) to the primary amine of a lysine residue of target protein under the synergistic action of UFM1 activating enzyme 5 (UBA5), UFM1-conjugating enzyme 1 (UFC1), [2] and UFM1 specific ligase (UFL1) [3]. UFM1, a highly conserved protein in diverse organisms, has a tertiary structure similar to ubiquitin. Upon C-terminal cleavage, the exposed glycine residue of UFM1 actively engages in subsequent cascade conjugating reactions [2].

To date, numerous studies have demonstrated that UFMylation is involved in many cellular processes, including endoplasmic reticulum (ER) homeostasis, ER-phagy [4], erythroid development [5], cell death and differentiation [6], and DNA damage response [7]. Consequently, UFMylation participates in the occurrence and development of various diseases, such as tumors and antiviral infections [8-12]. Ryosuke *et al*. reported that the UFM1 system induced ER-phage by Ufmylating NADH cytochrome b5 reductase 3 (CYB5R3), which is crucial for brain development [13]. Nevertheless, the role of the UFM1 system and UFMylation in neurological diseases remains not fully understood. In this study, we focus on the concept, cascade mechanism, capability, and potential role of UFMylation in neurological diseases.

## 2 ENZYMATIC CASCADE OF THE UFMYLATION SYSTEM

The enzymatic cascade of the UFMylation system is a key event that finely regulates protein function and cellular processes, involving pivotal components such as UFM1, UBA5, and UFL1. The UFM1 precursor (pro-UFM1) is a protein consisting of 85 amino acid residues with a molecular weight of approximately 9.1 kDa. Pro-UFM1 lacks biological activity until it undergoes cleavage at the C-terminus by UFM1-specific protease 1 (UFSP1) and UFSP2, exposing glycine residues at position 83 and ultimately generating mature UFM1 [2, 14]. UFSP1 is a cytoplasmic protein composed of 217 amino acid residues, while UFSP2 consists of 461 amino acid residues and is mainly located in the ER membrane. Both UFSP1 and UFSP2 are proteases that specifically cleave peptide bonds linked to the glycine residue of the C-terminal of UFM1, thereby facilitating the conversion of pro-UFM1 to mature UFM1 and the deufmylation of UFM1-substrate conjugates [15].

UBA5, composed of 404 amino acid residues, shares a highly homologous amino acid sequence at positions 72-229 with UBA1, which is a ubiquitin-specific activator enzyme. In the presence of ATP, UBA5 adenosylates glycine at position 83 of the C-terminus of UFM1, forming an intermediate complex through thioester bonds, thereby activating UFM1. Subsequently, the activated UFM1 is transferred to UFC1 through thioester bonds. UFC1, consisting of 167 amino acid residues, features a highly conserved region at positions 113-126 and contains an active site cysteine residue capable of forming thioester bonds.

**Fig. (1):**
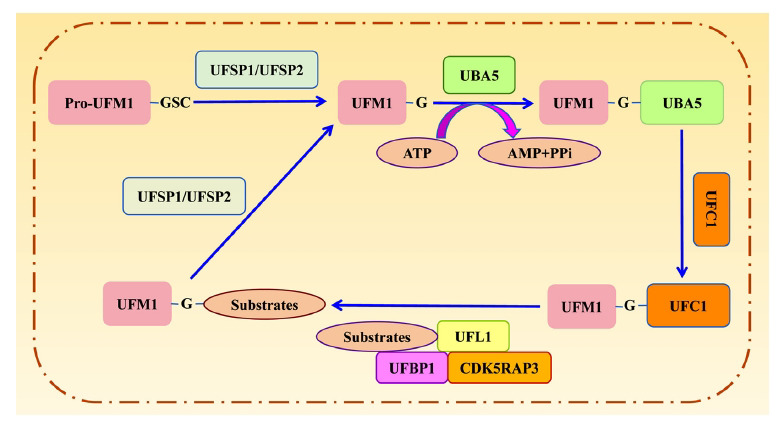
Overview of enzyme cascades in UFMylation system.

UFL1, consisting of 794 amino acid residues, interacts with UFC1 and substrates *via* the N-terminus α-helix, serving as the core region that promotes the combination of UFM1 to substrates [3, 16]. The activated UFM1 ultimately forms a covalent bond with the substrate through an E3 complex composed of UFL1, UFM1-binding and PCI domain-containing protein 1 (UFBP1, also named DDRGK1), and cyclin-dependent kinase 5 regulatory subunit-associated protein 3 (CDK5RAP3) (shown as Fig. **1**). Independently, UFC1 remains inactive; however, upon binding to UFBP1 and CDK5RAP3, it forms an active E3 ligase complex that triggers the activation of UFC1 for ammonolysis. Additionally, CDK5RAP3 acts as a substrate adapter, specifically regulating the UFMylation of the 60S ribosomal protein (RPL26), thereby contributing to ER homeostasis [17-19].

## 3 THE FUNCTIONS OF UFMYLATION

### 3.1 UFMylation and ER Protein Homeostasis

The maintenance of protein homeostasis encompasses a multitude of regulatory strategies, including the degradation pathways for misfolded proteins or translation-arrested products (APs). Ubiquitination of APs is induced by cytosol ribosome-associated quality control (RQC), promoting their degradation by the proteasome [20]. Misfolded proteins are transferred from the ER to the cytoplasm *via* ER-associated protein degradation (ERAD) [21]. In contrast to cytosol ERAD and RQC, the UFMylation of ribosomal proteins is implicated in ER-related RQC, facilitating the translocation of blocked ER proteins to lysosomes for degradation [19]. Activation of UFMylation of ribosomes, triggered by translation stalling during co-translational translocation, results in the specific and covalent binding of UFM1 to conserved lysine residues Lys132 and Lys134 at the C-terminus of RPL26. The UFMylation of RPL26 promotes the export of translocation-blocked proteins from ER to lysosomal for degradation. Additionally, confirmation studies also showed a close correlation between the UFMylation of RPL26 and the degradation of ER-APs. The RPL26 UFMylation participates in the degradation of ER APs under the synergistic effect of RQC [22].

CDK5RAP3 in the E3 complex acts as an adapter for RPL26 UFMylation, while another component, UFBP1, is associated with the 60S subunits of UFMylation RPL26, thereby involving in ER-RQC [23]. Moreover, UFBP1 is necessary for maintaining ER homoeostasis. UFMylation UFBP1 interacts with ER stress sensor inositol, requiring enzyme 1 (IRE1a) to regulate the stability of non-phosphorylated IRE1a, thereby maintaining ER homeostasis and reducing cell apoptosis [24]. HRD1 is also a target of UFM1 and normally interacts with UFL1 and UFBP1. Upon sensing ER stress, UFMylation HRD1 is involved in the degradation of misfolded proteins to maintain ER homeostasis [25]. However, further research is needed to investigate the specific process and mechanism by which RPL26 UFMylation promotes the degradation of translation-arrested proteins and ER-APs.

### 3.2 UFMylation and ER-phagy

Originally, UFM1 is bound to the shuffled ATG8 interacting motifs (sAIM) of cytoplasmic protein C53 [26]. Activated by ribosomal blockade during the process of co-translational protein translocation, C53 is recruited to the ER by forming complexes with UFL1 and ER membrane protein UFBP1. After binding to C53, UFM1 is subsequently transferred to RPL26, causing the exposure of sAIM in C53. The exposed sAIM then interacts with ATG8, mediating the autophagy degradation of newly generated stagnant peptide chains [27, 28]. The N-terminus of UFBP1 serves as the ER transmembrane domain, facilitating in its localization to the ER. Conversely, the C-terminus is the PCI domain that interacts with UFL1, recruiting UFL1 to the ER membrane. The interaction is pivotal for UFMylation and ER-phagy process [29]. Absence of UFBP1, as detected through isobaric tags for relative and absolute quantification based liquid chromatography-mass spectrometry, can affect the expression of proteins crucial for ER homeostasis, protein processing, and lysosome-related pathways. Moreover, UFBP1 deficiency impedes the fusion of autophagosomes and lysosomes, thereby inhibiting autophagic dissolution and increasing autophagosome accumulation, ultimately leading to cellular apoptosis [30]. However, the precise mechanism underlying UFBP1’s involvement in physiological activities has not been fully elucidated.

CYB5R3 is located on the ER membrane *via* its N-terminal myristoylation and membrane-binding domain. After covalent binding with UFM1 at lysine 214, the FAD binding domain of CYB5R3 is closed, leading to decreased enzyme activity. UFMylation CYB5R3 contributes to the activation of ER-phagy and is subsequently degraded by lysosomes under the dependence of CDK5RAP3. Deficiency in CYB5R3 UFMylation has been linked to microcephaly in mice, highlighting its significance in brain development [13]. However, the role and mechanisms of CDK5RAP3 in the degradation of UFMylation CYB5R3 need to be further explored.

### 3.3 UFMylation and DNA Damage Response

Under continuous stimulation from both internal and external environments, DNA undergoes various pathological changes, including DNA damage response (DDR). Following double-strand breakages (DSBs), the ataxia-telangiectasia mutated (ATM) kinase is activated by the MRE11-RAD50-NBS1 (MRN) complex and subsequently participates in downstream target protein phosphorylation [31]. During DNA damage, MRE11 is UFMylation at position K282. The MRE11 UFMylation promotes optimal ATM activation, which contributes to homologous recombination-mediated DSB repair and genome integrity [32]. Furthermore, during DSBs, UFL1 is recruited to the cleavage site by the MRN complex to promote the UFMylation of histone 4, thereby enhancing ATM activation. ATM, in turn, phosphorylates UFL1 at position S462, increasing UFL1 enzyme activity by forming a positive feedback loop [33]. This illustrates how UFMylation, particularly of MRE11 and histone 4 mediated by UFL1, critically optimizes ATM kinase activation during DNA damage.

On the contrary, UFSP2 inhibits DDR pathway activation during the occurrence of DSBs, maintaining association with the MRN complex in the absence of DNA damage. Upon encountering DSBs, UFSP2 undergoes ATM-mediated phosphorylation, resulting in its dissociation from the complex. After dephosphorylation by wild-type p53-induced phosphatase 1, UFSP2 is recruited to DSBs, promoting histone 4 to deUFMylation and dampening ATM activation, thus establishing a negative feedback loop [34]. The alternating regulation of positive and negative feedback ensures appropriate DDR, which is crucial for maintaining genetic integrity. Improper DDR can cause genomic instability, premature aging, and the onset of conditions such as cancer and neurodegenerative diseases [35].

## 4 UFMYLATION AND NEUROLOGICAL DISEASES

UFMylation is a regulatory model of PTMs that has been proven to be associated with the occurrence and development of various diseases. Key components such as UBA5, UFL1, and UFBP1 are vital for embryonic hematopoiesis and erythroid development. Deficiency in the UFMylation system can cause DNA damage and cell death in hematopoietic stem cells [5, 6, 36]. Additionally, the UFMylation system regulates the production of inflammatory factors, enhances antiviral infection resistance, and inhibits virus amplification [11, 37-40]. Extensive studies have shown the close association between the UFMylation system and tumorigenesis and drug resistance [8, 9, 41-43]. Besides, emerging evidence has linked the UFMylation system to the pathogenesis of neurological diseases. In the present study, we focus on discussing the role of the UFMylation system in various neurological diseases, aiming to provide direction for future research.

### 4.1 UFMylation and Epileptic Encephalopathy

Epileptic encephalopathy, characterized by frequent epileptic seizures or discharges, leads to progressive brain dysfunction and acquired chronic neurological decline, predominantly affecting newborns, infants, and children. Polymorphisms in several genes encoding proteins involved in the UFMylation pathway have been reported to be associated with the risk of epileptic encephalopathy. For example, pathogenic compound heterozygous variant in *UBA5* (c.1111G>A (p.Ala371Thr)) was found in infants with severe epilepsy syndrome, often accompanied by a missense mutation c.164G>A (p.Arg55His) or nonsense mutations, such as c.562C>T (p.Arg188Ter), c.855C>A (p.Tyr285Ter), and c.181C>T (p.Arg61Ter). Functional analyses of the variants suggest that reduced UBA5 enzyme activity, leading to UFMylation system deficiency, underlies the development of the syndrome [44].

Pathogenic complex heterozygous genotypes were also detected in the *UBA5* gene by sequencing and comparative analyzing the genomes of a pair of sisters with early-onset epileptic encephalopathy and their unaffected parents. One mutation, c.684G > A (p. Ala228=), occurs at the last nucleotide position of exon 7 of the *UBA5* gene, representing an exonic splicing mutation inherited from the father. Another mutation, c.1111G > A (p.Ala371Thr), inherited from the mother, has not yet been previously confirmed to be related to neurological disorders when occurring as a single homozygous mutation. It appears to be pathogenic only in the context of forming complex heterozygous genotypes with other mutations [45].

Additionally, the mutation c.1111G > A (p.Ala371Thr) was discovered in early-onset epilepsy patients from four other families, alongside additional mutations: one with c.562C>T (p.Arg188Ter), two with c.907T>C (p.Cys303Arg), and a pair of siblings with c.761T>C (p.Leu254Pro) [46]. To investigate the influence of various mutants on the UFMylation process, *UBA5^C303R^*, *UBA5^L254P^*, and *UBA5^A371T^* mutants were introduced into experiments involving UFM1 and UBA5 intermediate formation. Results showed that the *UBA5^C303R^* and *UBA5^L254P^* mutants impaired the formation of UFM1 and UBA5 complexes, suggestive of reduced UBA5 enzyme activity due to the mutations. On the contrary, the *UBA5^A371T^* mutant formed an intermediate with UFM1 but failed to transfer the activated UFM1 to UFC1 [46].

Previously, patients with early-onset epilepsy were found to harbor various mutations in the *UBA5* gene, primarily presenting as compound heterozygous mutations. However, an isolated homozygous mutation, c.158A > T (p.Tyr53Phe), resulting in a decrease in UBA5 transthiolation activity to 6.8%, was identified in a sporadic case of early myoclonic epilepsy or Aicardi syndrome [47]. Moreover, a microdeletion of approximately 3.2 Mb (Chr3: 129762317-132948291) within the 3q22.1 region, leading to loss of *UBA5* gene expression, and a hemizygous missense mutation, *UBA5* c.214C > T (p.Arg72Cys), were observed in the genome of a child with persistent West syndrome, epileptic spasms, severe developmental delay, severe encephalopathy, and cerebellar atrophy [48].

The *UFSP2* variants have also been implicated in epilepsy risk. Exome sequencing of three patients with severe early-onset neurological disorders and epilepsy within a family revealed a homozygous mutation of c.344T > A (p.Val115Glu) in the *UFSP2* gene, while their parents were heterozygous carriers. Functional analysis showed that the c.344T > A mutation induced a reduction in UFSP2 protein stability, resulting in lower levels of UFSP2 and perturbation of de-Ufmyaltion processes [49].

*In vivo* experiments in mice showed that heterozygous mutants in the *UFBP1* gene exhibited abnormal electroencephalograms, leading to spontaneous and recurrent epileptic events [50]. Pan *et al*. investigated the relationship between the genotype and phenotype of *UBA5* gene mutations in developmental and epileptic encephalopathy-44. By comparing survival rates, developmental time, lifespan, and motor abnormalities among flies with different *UBA5* gene mutations, alongside assessing UBA5 protein stability, ATP binding capacity, UFM1 activation, and UFM1 transthiolation through separately *in vivo* and *in vitro* experiments, the results showed that mutations in *UBA5* destroyed enzyme activity, leading to clinical manifestations of varying severities. Notably, lower UBA5 enzyme activity was accompanied by more severe clinical symptoms [51].

**Fig. (2):**
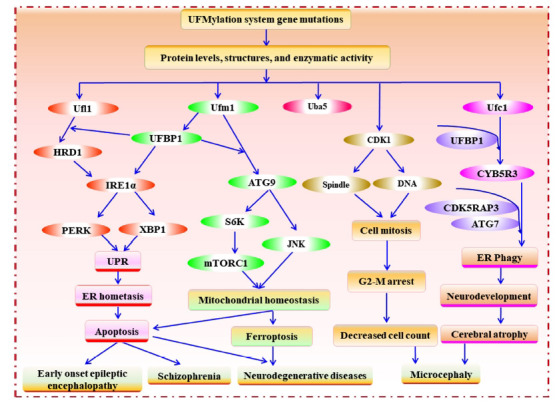
Overview of the role of the UFMylation system in the pathogenesis of neurological diseases.

As mentioned previously, the ER stress sensor protein IRE1α, crucial for ER homeostasis, has been proven to be closely related to the occurrence of epileptic encephalopathy [52-54]. Knockdown of UFM1 attenuated IRE1α levels by disrupting the UFMylation of UBFP1. Consequently, IRE1α-X-box-binding protein 1 (XBP1) signal transduction was inhibited, accompanied by activation of the pancreatic ER kinase-like ER kinase (PERK) pathway, ultimately leading to cell apoptosis. Activation of the PERK-unfolded protein response (UPR) pathway disrupted ER homeostasis and triggered apoptosis, thereby promoting epileptogenesis [24] (shown as Fig. **2**). These findings underscore the critical role of genetic variations in the UFMylation pathway in contributing to the pathogenesis of epileptic encephalopathy.

### 4.2 UFMylation and Microcephaly

Growing evidence suggests that mutations in genes associated with the UFMylation pathway contribute to the susceptibility to microcephaly. Whole exome sequencing (WES) unveiled rare biallelic mutations in *UBA5* among five children from four different families, all of whom suffered from early-onset encephalopathy accompanied by microcephaly. These mutations included five missense mutations: c.1165G > T (p. Asp389Tyr), c.778G > A (p.Val260Met), c.1111G > A (p.Ala371Thr), c.169A > G (p.Met57Val), c.503G > A (p.Gly168Glu), one insertional frameshift mutation c.971_972insC (p.Lys324Asnfs*14), and one nonsense mutation c.904C > T (p.Gln302Ter). Each affected individual carried compound heterozygous mutations rather than a singular mutation [55]. Functional analysis assessing the effects of these seven mutations on UFM1 activation and UFM1-UFC1 conjugation indicated that mutations c.503G > A and c.904C > T completely abolished UBA5 enzyme activity, while mutations c.778G > A, c.971_972insC, and c.169A > G reduced enzyme activity to varying extents. Surprisingly, mutations c.1165G > T and c.1111G > A retained enzyme activity. Additionally, in a patient with acquired microcephaly, motor disorders, and neurodevelopmental delay, a 597 kb deletion resulting in complete *UBA5* deletion on chromosome 3q22.1 was detected, alongside a hemizygous missense mutation c.1166A G (p.Asp389Gly) on another chromosome [56]. Apart from *UBA5*, homozygous variation of *UFM1* (c.241C > T (p: Arg81Cys) and *UFC1* (c.317C > T (p: Thr106Ile), c.68G > A (p: Arg23Gln)) were also identified in multiple children with progressive microcephaly [57].

The UFMylation system influences various physiological processes implicated in microcephaly onset. Knockdown of genes associated with UFMylation, such as UFM1, UBA5, UFC1, or UFL1, in *Drosophila* neuroblasts resulted in diminished brain size due to a decrease in the neuroblast population. The reduction of neuroblasts was correlated to impaired cell mitosis. Specifically, defective UFMylation system function led to an increased proportion of cells in the G2/M phase, accompanied by disrupted spindle and DNA arrangement positions. The deficiency of UFL1 prompted a shortened cell synthesis phase and premature entry into mitosis, resulting in aberrant spindle morphology and chromosomal segregation. Moreover, the UFMylation system regulated cell cycle transition by modulating the phosphorylation level of cyclin-dependent kinase 1 [58] (shown in Fig. **2**).

UFL1 and UFBP1 were confirmed to play a crucial role in the survival of mouse neurons, as the *UFL1* and *UFBP1* knockout in mice led to a significant decrease in brain protein levels, increased neuronal cell death in the cortex and hippocampus, ultimately resulting in microcephaly [50]. With a dependency on UFL1 and UFBP1, CYB5R3 was UFMylated to induce ER-phagy under the mediation of CDK5RAP3 and ATG7 and contribute to neural development. Insufficient UFL1 and UFBP1 levels resulted in UFMylation defects in CYB5R3, thereby promoting microcephaly [13]. Mutations in *CYB5R3* have been associated with congenital methemoglobinemia type II, a condition linked to microcephaly [59, 60]. It is speculated that UFMylation-defective CYB5R3 may interfere with the ER-phagy or trigger congenital methemoglobinemia type II, thereby affecting nervous system development and contributing to the onset of neurological diseases.

### 4.3 UFMylation and Neurodegenerative Diseases

Neurodegenerative diseases are caused by progressive loss of neurons or their medullary sheaths, ultimately leading to functional impairment. Genetic mutations in the UFMylation system have also been shown to be associated with the pathogenesis of neurodegenerative diseases. Autosomal recessive cerebellar ataxia (ARCA) represents a broadly heterogeneous neurodegenerative condition. WES identified two heterozygous mutations, *UBA5* c.568C > T (p. Arg246Ter) and c.760A > G (p. Lys310Glu), in the genomes of two ARCA patients. Functional analysis revealed that the c.568C > T mutation led to a complete loss of UFM1 enzyme activity, whereas the c.760A > G mutation exhibited reduced stability but retained the ability to interact with UFM1, albeit with weakened enzyme activity [61].

Hypomyelination with atrophy of the basal ganglia and cerebellum (H-ABC) is a neurodegenerative disease characterized by delayed neural development, motor and speech degeneration, and rare leukodystrophy. Through WES coupled with Sanger analysis, a homozygous mutation, c.- 273_ -271delTCA, was discovered within the promoter region of *UFM1* in H-ABC patients without negative tubulin beta 4A gene mutations [62]. Subsequent investigation from a single center survey revealed that H-ABC induced by *UFM1* c.-273_-271delTCA mutation was accompanied by severe early encephalopathy with hissing and hearing and visual impairment [63]. In a separate case involving hypomyelination and atrophy, WES identified a *UBA5* homozygous mutation, c.895C>T (p: Pro299Ser), which caused significantly reduced enzyme activity [64]. Furthermore, the *UFM1* c. -155_ -153delTCA mutation was found in patients with hypomyelinating leukodystrophy, a neurodegenerative disorder, exerting the impairment of transcriptional activity [65].

Notably, the transcription levels of UFMylation pathway genes in the brain tissue of older *Drosophila* were lower compared to those in younger counterparts [66]. Knockdown experiments targeting key genes of the UFMylation system, such as *UBA5*, *UFL1*, and *UFBP1*, yielded a significant increase in neuronal apoptosis, indicating a potential neuroprotective effect of the UFMylation system. Deficiencies in UFMylation were found to impair lysosomal autophagy function. ATG9, a member of the autophagy protein family, was identified as a substrate for UFM1 covalent conjugation, a process disrupted by the absence of UFBP1. The UFMylation of ATG9 diminished the mechanistic target of rapamycin complex 1 activity by increasing S6K phosphorylation, exerting a protective effect on the degenerative brain. Moreover, the UFMylation system can also exhibit neuroprotective effects by regulating mitochondrial homeostasis and c-Jun N-terminal kinase activity through ATG9 (shown as Fig. **2**). Considering that mitochondrial dysfunction and oxidative stress are recognized contributors to neurodegenerative diseases [67-69], it is imperative to elucidate the precise mechanisms by which the UFMylation system influences mitochondrial function and regulates cellular processes such as apoptosis and ferroptosis [67, 70]. Understanding these mechanisms may offer novel insights into the pathogenesis and therapeutic avenues for neurodegenerative diseases.

### 4.4 UFMylation and Schizophrenia

Schizophrenia is a heterogeneous psychiatric disorder characterized by emotional and behavioral abnormalities accompanied by emotional and cognitive confusion. In a study involving 13 pairs of schizophrenia patients and matched controls, cadaveric dissection was conducted to obtain gray matter samples from the left superior temporal gyrus cortex, each ranging from 0.8 to 1 cm^3^ in volume. Following tissue homogenization and extraction, the expression of diverse target proteins associated with the UFMylation system was detected by using Western blotting analysis. The results showed that the UFL1 levels were decreased in the superior temporal gyrus of schizophrenia patients, while the levels of UBA5 and UFC1 remained invariable [71]. These findings indicate a diminution of UFMylation levels in the brain tissue of schizophrenia patients.

In 2018, Kim *et al*. detected abnormal expression of proteins involved in endoplasmic reticulum quality control and ERAD in the brain tissue of patients with schizophrenia, showing an upregulation of HRD1 expression [72]. HRD1, a pivotal ubiquitin ligase in ERAD, forms a protein transduction pathway through autoubiquitination, which facilitates the translocation of misfolded proteins from the ER to the cytoplasm for degradation by proteasomes [73]. The proper functioning of the ERAD pathway necessitates the UFMylation-mediated autoubiquitination of HRD1. Disruption of HRD1 UFMylation resulted in increased levels of p-PERK, PERK, and XBP1, thereby activating the UPR process, perturbing the ER homeostasis, and inducing cell apoptosis [25] (shown as Fig. **2**). ER stress and activation of the UPR pathway have been demonstrated to be a crucial contributor in the pathogenesis of schizophrenia. Taken together, previous results indicate that the lack of UFMylation components diminishes the UFMylation HRD1, activates UPR to regulate ER protein homeostasis, and thus induces the pathogenesis of schizophrenia [52, 74]. These insights provide valuable information in identifying novel therapeutic targets for schizophrenia associated with UFMylation pathways.

### 4.5 UFMylation and Neuropathy

A recent study has reported that mutation in *UBA5* can precipitate fatal congenital neuropathy. Within a consanguineous family, several infants presented with congenital peripheral motor neuropathy mainly characterized by manifestations such as irritability, limb weakness, and severe motor developmental delays. Despite normal findings on brain and spinal magnetic resonance imaging as well as electroencephalography, these infants died from respiratory failure between 19 days and 16 weeks postpartum. WES and linkage analysis identified a homozygous variation of c.31C>T (p. Arg11Trp) in *UBA5* among all affected individuals. The mutation can not only lead to diminished levels of UBA5 protein but also compromise its ability to activate UFM1 [75]. However, further investigations are warranted to elucidate the precise pathological mechanisms underlying this phenomenon.

## CONCLUSION

During the past decades, various types of PTMs have been observed in proteins, such as phosphorylation, acetylation, methylation, ubiquitination, and UFMylation. UFMylation, a recently recognized PTM, plays a pivotal role in numerous cellular processes, including ER homeostasis, ER-phagy, and DNA damage response, thereby contributing to the initiation and progression of a spectrum of disorders such as tumors, infections, and hematological disorders. Here, the potential role of UFMylation in neurological diseases was discussed, with gene mutations in the UFMylation system influencing the risk of epileptic encephalopathy, microcephaly, neurodegenerative diseases, and schizophrenia (Table **1**). However, current evidence is preliminary, and many unresolved issues remain to be addressed. How does the UFMylation system regulate IRE1α stability to ensure ER homeostasis? Which pathway is involved in the ER-phagy process by UFMylation CYB5R3? How does the UFMylation system regulate mitochondrial function to participate in apoptosis and ferroptosis in neurodegenerative diseases? How does the deficiency of HRD1 UFMylation disrupt ER homeostasis and activate UPR? Despite these unanswered questions, previous findings serve as a catalyst for opening new avenues of research in the future. Addressing these gaps not only enhances our understanding of the role of the UFMylation system in neurological disorders but also provides opportunities to explore novel therapeutic strategies.

## Figures and Tables

**Table 1:** Mutations in the UFMylation system associated with neurological disorders.

**Genes**	**Variants**	**Protein Changes**	**Physiology**	**Pathology**	**References**
*UFM1*	c.241C > T	Arg81Cys	1. Reduce UFM1-UBA5 intermediates 2. Almost no UFM1-UFC1 intermediates	Early-onset encephalopathy with progressive microcephaly	[57]
	c.- 273_ -271delTCA	/	Reduce promoter activity in specific CNS cell lines	Hypomyelination with atrophy of the basal ganglia and cerebellum	[62, 63]
	c. -155_ -153delTCA	/	Reduce transcriptional activity	Hypomyelinating leukodystrophy	[65]
*UBA5*	c.1111G > A	Ala371Thr	1. Almost no UFM1 conjugates 2. Reduce the ability to transfer activated UFM1 to UFC1 3. Reduce E2 activity but not E1 activity	Early epileptic encephalopathy	[44-46, 55]
	c.164G > A	Arg55His	4. Reduce UBA5 mRNA and protein level 5. Almost no UFM1 conjugates 6. Reduce ability to form UFM1-UBA5 intermediates 7. Reduce the ability to transfer activated UFM1 to UFC1		[44]
	c.562C > T	Arg188Ter	Compound heterozygous mutation		[44, 46]
	c.855C > A	Tyr285Ter	Compound heterozygous mutation		[44]
	c.181C > T	Arg61Ter	Compound heterozygous mutation		[44]
	c.684G > A	Ala228=	Exonic splicing mutation		[45]
	c.158A > T	Tyr53Phe	1. The transcription level is basically not affected 2. Transthiolation activity reduced to 6.8%		[47]
	c.907T > C	Cys303Arg	3. Reduce ability to form UFM1-UBA5 intermediates 4. No UFM1 activation 5. Reduce the ability to transfer UFM1 to UFC1		[46]
	c.761T > C	Leu254Pro			
	A microdeletion of approximately 3.2Mb in the 3q22.1 (Chr3: 129762317-132948291)		UBA5 expression deficiency	Intractable West syndrome	[48]
	c.214C > T	Arg72Cys	A hemizygous missense variant		
	c.1165G > T	Asp389Tyr	Barely reduce catalytic activity	Severe infantile-onset encephalopathy accompanied by epilepsy and microcephaly	[55]
	c.778G > A	Val260Met	1. Delay trans-thiol activity 2. Reduce catalytic activity		
	c.169A > G	Met57Val	3. Delay trans-thiol activity 4. Reduce catalytic activity		
	c.503G > A	Gly168Glu	5. Reduce ability to form UFM1-UBA5 intermediates 6. No trans-thiol activity 7. No catalytic activity		
	c.971_972insC	Lys324Asnfs*14	8. No trans-thiol activity 9. Reduce catalytic activity		
	c.904C > T	Gln302Ter	10. Reduce ability to form UFM1-UBA5 intermediates 11. No trans-thiol activity 12. No catalytic activity		
	c.1166A > G	Asp389Gly	Structural changes of the C-terminal region of UBA5	Severe neurodevelopmental delay with acquired microcephaly	[56]
	c.568C > T	Arg246Ter	1. Reduce stability of UBA5 2. Almost no UFM1-UBA5 intermediates	Autosomal recessive cerebellar ataxia	[61]
	c.760A > G	Lys310Glu	Reduce stability of UBA5		
	c.895C > T	Pro299Ser	1. Delay trans-thiol activity 2. Reduce the ability to transfer UFM1 to UFC1	Hypomyelination with thalamic involvement and axonal neuropathy	[64]
	c.31C > T	p. Arg11Trp	1. Reduce UBA5 protein 2. Lower ability to activate UFM1	Fatal congenital neuropathy	[75]
*UFC1*	c.317C > T	Thr106Ile	Few UFM1-UBA5 intermediates	Early-onset encephalopathy with progressive microcephaly	[57]
	c.68G > A	Arg23Gln			
*UFS2*	c.344T > A	Val115Glu	1. Reduce UFSP2 protein level but not mRNA 2. Destruction of UFSP2 structure	Neurodevelopmental disability and epilepsy	[49]

## LIST OF ABBREVIATIONS

AP: Arrested Product
ARCA: Recessive Cerebellar Ataxia
ATM: Ataxia-telangiectasia Mutated
CDK5RAP3: Cyclin-dependent Kinase 5 Regulatory Subunit-associated Protein 3
CYB5R3: Cytochrome b5 Reductase 3
DDR: DNA Damage Response
DNA: Deoxyribonucleic Acid
DSB: Double-strand Breakage
ER: Endoplasmic Reticulum
ERAD: ER-associated Protein Degradation
H-ABC: Hypomyelination with Atrophy of the Basal Ganglia and Cerebellum
HRD1: HMG-CoA Reductase Degradation Protein
IRE1a: ER Stress Sensor Inositol Requiring Enzyme 1
MRN: MRE11-RAD50-NBS1 Complex
PERK: Pancreatic ER kinase-like ER Kinase
PTM: Post-translational Modification
RPL26: 60S Ribosomal Protein
RQC: Ribosome-associated Quality Control
sAIM: Shuffled ATG8 Interacting Motifs
UBA5: UFM1 Activating Enzyme 5
UFBP1: UFM1-binding and PCI Domain-containing Protein 1
UFC1: UFM1 Conjugating Enzyme 1
UFL1: UFM1 Specific Ligase 1
UFM1: Ubiquitin-fold Modifier 1
UFSP1: UFM1-specific Protease 1
UFSP2: UFM1-specific protease 2
WES: Whole Exome Sequencing
XBP1: X-box-binding Protein 1
